# The role of ribonucleases in regulating global mRNA levels in the model organism *Thermus thermophilus* HB8

**DOI:** 10.1186/1471-2164-15-386

**Published:** 2014-05-19

**Authors:** Hiromasa Ohyama, Tomofumi Sakai, Yoshihiro Agari, Kenji Fukui, Noriko Nakagawa, Akeo Shinkai, Ryoji Masui, Seiki Kuramitsu

**Affiliations:** 1 Department of Biological Sciences, Graduate School of Science, Osaka University, 1-1 Machikaneyama-cho, Toyonaka, Osaka 560-0043, Japan; 2 Graduate School of Frontier Biosciences, Osaka University, 1-3 Yamadaoka, Suita, Osaka 565-0871, Japan; 3 RIKEN SPring-8 Center, 1-1-1 Kouto, Sayo, Hyogo 679-5148, Japan; 4 RIKEN Structural Biology Laboratory, 1-7-22, Suehiro-cho, Tsurumi-ku, Yokohama, Kanagawa 230-0045, Japan

**Keywords:** RNase, Transcriptome analysis, Whole-cell research

## Abstract

**Background:**

RNA metabolism, including RNA synthesis and RNA degradation, is one of the most conserved biological systems and has been intensively studied; however, the degradation network of ribonucleases (RNases) and RNA substrates is not fully understood.

**Results:**

The genome of the extreme thermophile, *Thermus thermophilus* HB8 includes 15 genes that encode RNases or putative RNases. Using DNA microarray analyses, we examined the effects of disruption of each RNase on mRNA abundance. Disruption of the genes encoding RNase J, RecJ-like protein and RNase P could not be isolated, indicating that these RNases are essential for cell viability. Disruption of the TTHA0252 gene, which was not previously considered to be involved in mRNA degradation, affected mRNA abundance, as did disruption of the putative RNases, YbeY and PhoH-like proteins, suggesting that they have RNase activity. The effects on mRNA abundance of disruption of several RNase genes were dependent on the phase of cell growth. Disruption of the RNase Y and RNase HII genes affected mRNA levels only during the log phase, whereas disruption of the PhoH-like gene affected mRNA levels only during the stationary phase. Moreover, disruption of the RNase R and PNPase genes had a greater impact on mRNA abundance during the stationary phase than the log phase, whereas the opposite was true for the TTHA0252 gene disruptant. Similar changes in mRNA levels were observed after disruption of YbeY or PhoH-like genes. The changes in mRNA levels in the bacterial Argonaute disruptant were similar to those in the RNase HI and RNase HII gene disruptants, suggesting that bacterial Argonaute is a functional homolog of RNase H.

**Conclusion:**

This study suggests that *T. thermophilus* HB8 has 13 functional RNases and that each RNase has a different function in the cell. The putative RNases, TTHA0252, YbeY and PhoH-like proteins, are suggested to have RNase activity and to be involved in mRNA degradation. In addition, PhoH-like and YbeY proteins may act cooperatively in the stationary phase. This study also suggests that endo-RNases function mainly during the log phase, whereas exo-RNases function mainly during the stationary phase. RNase HI and RNase HII may have similar substrate selectivity.

## Background

All organisms use genetic information during DNA replication, transcription and translation. This process is known as the central dogma, where the decoding of genetic information during gene transcription and protein synthesis is mediated by RNA. In addition, proteins levels are controlled, in part, by those of RNA. Thus, the control of RNA abundance is crucially important. Many studies have investigated sigma factors and transcriptional regulators, which are involved in RNA synthesis. However, RNA levels are controlled by RNA degradation, as well as RNA synthesis. Thus, studies of RNA degradation are also necessary to understand RNA levels. Ribonucleases (RNases) are the main enzymes in RNA degradation and maturation pathways, and they have been widely studied. However, the overall degradation network of RNases and the substrates of this degradation have not been fully elucidated at the whole cell. This is partly because RNases are considerably smaller in number than substrate RNAs. In humans, it is believed that there are approximately 25,000 total genes [1], of which fewer than 100 are RNase genes. Thus, a single RNase must degrade hundreds or thousands of RNAs and must therefore have broad substrate specificity. It is necessary to determine these substrate specificities and to develop a comprehensive view of the RNA degradation system in the whole cell.

The contribution of each RNase to RNA degradation has not been fully delineated, even for well-known RNases, particularly in terms of the functional interrelationships between these enzymes. Some studies have investigated the global control of RNA turnover, but these studies were focused on endo-RNases, which are believed to degrade mRNA [2,3]. Many substrates have been identified for each exo-RNase [4], but no endogenous substrates have been assignment of to these enzymes based on global substrate profiling. In addition, RNases that were considered not to be involved in mRNA decay were excluded from these investigations. Moreover, recent advances in genome analysis have led to the identification of several RNases of functionally unknown. For example, the β-CASP family proteins were identified as 5′–3′ exo-RNases [5,6] although their physiological function is unknown.

The extreme thermophile *Thermus thermophilus* HB8 is a Gram-negative eubacterium, which can grow at temperatures over 75°C [7]. We selected *T. thermophilus* HB8 as a model organism for the present study for several reasons: (i) it has a smaller genome size than other model organisms; (ii) each biological system in *T. thermophilus* is comprises of only essential enzymes, (iii) *T. thermophilus* HB8 is one of the most widely analyzed organisms in terms of structural analysis. In addition, the regulation of RNA synthesis in *T. thermophilus* HB8 was studied by Shinkai *et al*., [8-10]; therefore, we hoped to gain insight into the relationship between RNA synthesis and degradation. According to the BLAST research, 15 putative RNases have been identified in *T. thermophilus* HB8 genome (Table 1). RNase Y is a key component of the RNA degradosome of *Bacillus subtilis* along with RNase J1/J2, PNPase and several other enzymes [11,12]. RNase J1 is a dual-function RNase, which possesses both 5′–3′ exo-RNase and endo-RNase activity [5,13]. Indeed, RNase J1 was the first example of 5′–3′ exo-RNase in bacteria [5]. PNPase and RNase R degrade RNA fragments generated by endo-RNases. *T. thermophilus* lacks RNase E, which is an essential endo-RNase in *Escherichia coli*, whereas it possesses RNase Y and RNase J, suggesting that its RNA degradation pathways are similar to those of *B. subtilis* (Additional file 1). RNase Y and RNase J cleave mRNA endonuclotically and produce RNA fragments that are degraded exonucleotically by PNPase and RNase R. Finally, nanoRNase degrades small RNA fragments [14]. In addition to these proven RNases, *T. thermophilus* HB8 has several putative RNases, such as Argonaute and L-PSP.

**Table 1:** **Putative RNases of *Thermus thermophilus *** **HB8**

**Protein name**	**ORF ID**	**Accession number**	**Major substrate**	**Activity**	**Reference**	***B. subtilis***	***E. coli***
RNase J	TTHA1140	YP_144406.1	mRNA	Endo & Exo	[5,15]	○ (40%)	×
RNase Y	TTHA1817	YP_145083.1	mRNA	Endo	[16]	○ (49%)	×
RNase II	TTHA1534	YP_144800.1	mRNA	Exo	[17]	×	○
RNase R	TTHA0910	YP_144176.1	mRNA	Exo	[17]	○ (31%)	○ (26%)
PNPase	TTHA1139	YP_144405.1	mRNA	Exo	[17]	○ (49%)	○ (46%)
RNase HI	TTHA1556	YP_144822.1	DNA/RNA hybrid	Endo	[18]	×	○ (50%)
RNase HII	TTHA0198	YP_143464.1	DNA/RNA hybrid	Endo	[18]	○ (38%)	○ (40%)
RNase P*	TTHA0445	YP_143711.1	tRNA precursor	Endo	[19]	○ (22%)	○ (18%)
Argonaute	TTHB068	YP_145307.1	Phage RNA	Endo	[20]	×	×
RecJ-like protein	TTHA0118	YP_143384.1	Short RNA	Exo	[21]	○ (21%)	×
β-CASP family protein	TTHA0252	YP_143518.1	mRNA?	Exo	[6]	×	×
YbeY	TTHA1045	YP_144311.1	rRNA precursor?	Endo	[16]	○ (29%)	○ (21%)
PhoH-like protein	TTHA1046	YP_144312.1	?	??	[22]**	○ (50%)	○ (47%)
L-PSP	TTHA0137	YP_143403.1	?	Endo	[23]	○ (50%)	○ (37%)
PIN-domain protein	TTHA0540	YP_143806.1	?	??	[24,25]	○ (29%)	×

The circles and crosses indicate the presence and absence of an ortholog, respectively.

The numbers in parentheses are the percentage amino acid sequence identity of that protein with its homologs.

*The catalytic unit of RNase P is RNA.

**Predicted by sequence similarity without experimental evidence.

In this study, transcriptome analyses were performed with DNA microarrays to obtain information about potential RNA substrates of RNases. Our analysis showed that mRNA abundance was affected by disruption of known RNases, as well as by putative RNases that were not previously considered to be involved in mRNA decay. The effects of disruption of several RNases were influenced by the growth phase and the types of genes affected were different among three 3′–5′ exo-RNases, which have similar activity. In addition, the disruption of certain RNases caused similar changes in mRNA abundance. These results suggest the existence of cooperative and functional overlaps among the RNases in *T. thermophilus*.

## Results

### Putative RNases in the *T. thermophilus* HB8 genome

To identify RNase genes in *T. thermophilus* HB8, we analyzed the genome sequence based on sequence similarity and we also searched the literature. As a result, we identified 15 putative RNases genes (Table 1). BLAST searches showed that TTHA1140, TTHA1817, TTHA1139, TTHA0198, TTHA1556 and TTHA0445 shared a high level of sequence similarity with RNase J, RNase Y, PNPase, RNase HII (type II RNase H), RNase HI (type I RNase H) and RNase P, respectively. TTHA1140 is an ortholog of TTC0775, which was shown to be RNase J [5]. TTHA1556 is an ortholog of RNase HI, which degrades DNA/RNA hybrids. TTHA0198 is an ortholog of RNase HII, which is known to cleave RNA-DNA junctions only at the 5’ side of the junction [26]. RNase II and RNase R share the same basic structural organization: a central catalytic RNB domain, two N-terminal cold shock domains and one C- terminal S1 domain. The homology scores allowed us to annotate TTHA1534 and TTHA0910 as RNase II and RNase R, respectively. *T. thermophilus* HB8 Argonaute, an RNase component of the RNA-induced silencing complex in eukaryotes, was assigned as TTHB068 because the ortholog of *T. thermophilus* HB27 (TT_P0026), with only two substitutions, was shown to be Argonaute [20]. Previously, we showed that TTHA0118 and TTHA0252 degrade RNA [6,21]. TTHA0118 specifically degrades short oligo RNAs (“nanoRNA”), and is therefore referred to as a nanoRNase [27]. TTHA0252 belongs to the β-CASP family of the metallo-β-lactamase superfamily, of which RNase J is also a member. The tertiary structures of these proteins are similar except for the C-terminal domain [6,13].

In addition to these known RNases, we predicted that several further RNase candidates. TTHA1046 possesses a PhoH-like motif, which is predicted to comprise an ATPase domain and an NYN ribonuclease domain [22]. In addition, the N-terminal sequence of TTHA1046 appears to have a KH motif, which is a well-known RNA-binding motif. Therefore, we predicted that TTHA1046 is a putative RNase. The *E. coli* ortholog of TTHA1045, YbeY, may be involved with rRNA maturation based on mutagenic studies and its strong genetic interactions with PNPase and RNase R [28]. Furthermore, there is tertiary structural similarity between YbeY and the MID domain of Argonaute [29]. Thus, we predicted that TTHA1045 is a candidate RNase. TTHA0137 belongs to the endo-RNase L-PSP family. The name of this family originates from rat liver perchloric acid-soluble protein, which exhibits RNase activity [23]. TTHA0540 has a PIN domain motif, which is known to be an RNase domain [24,25]. Thus, we predicted that TTHA0137 and TTHA0540 are candidate RNases.

It should be noted that some toxin components of the toxin–antitoxin system have RNase activities [30]. The genome of *T. thermophilus* HB8 contains more than 20 pairs of genes that code for toxin–antitoxin components, some of which contain a PIN domain motif. In addition, some CRISPR-associated (Cas) proteins are known to possess RNase activities [31]. A Cas protein from *T. thermophilus* HB8, Cse3, has already been reported [32]. However, we excluded putative RNases in the toxin-antitoxin system and CRISPR-mediated immune system from this study on the basis that these RNases do not degrade mRNAs under normal growth conditions.

### Disruption of RNase genes

To uncover the functions of *T. thermophilus* RNases *in vivo*, we successfully disrupted 12 of the 15 putative *T. thermophilus* HB8 RNase genes. Gene disruption was performed by homologous recombination using a thermostable kanamycin-resistant marker. Despite repeated attempts, no kanamycin-resistant colonies were obtained after disrupting RNase J, RNase P or nanoRNase. These results suggest that each of these three genes is essential for the growth of *T. thermophilus* HB8 under the conditions used in this study. It is perhaps to be expected that the protein component of RNase P is essential for cell viability because this enzyme catalyzes 5’-end maturation of tRNA species, which is a vital process during the production of mature tRNAs. The protein subunit of RNase P is essential for the cleavage of tRNA precursors in normal growth conditions [33], and it is an essential gene in *B. subtilis*[34]. Thus, disruption of the TTHA0445 gene, which encodes the protein component of RNase P, may result in the deletion of RNase P. The gene that encodes RNase J has been reported to be essential in *B. subtilis* and *Streptococcus pyogenes*[35]. However, it should be noted that a *Sinorhizobium meliloti* disruptant of the single RNase J gene was viable [36]. NanoRNase is believed to be a functional homologue of *E. coli* oligoribonuclease (Orn), which is essential for cell viability [37]. However, we previously reported the disruption of the nanoRNase gene, TTHA0118 [21]. This might be due to the polyploid nature of *T. thermophilus* HB8 genome (as discussed later). Except for RNase R, deletions of the other 12 RNase genes did not affect the cellular growth rate.

### DNA microarray analysis of RNase disruptants

To identify the role of each RNase in the regulation of global mRNA abundance, we performed transcriptome analyses of the RNase disruptants using DNA microarrays. We grew cultures in rich medium and analyzed wild-type and disruptant cells at the log (exponential growth phase) and stationary phases.

The overall mRNA levels of each disruptant were analyzed at the ORF level and compared with those of the wild-type cells. The genes were screened based on a false discovery rate (<0.05) and a fold change (>2 or < 0.5) relative to the wild type. These results are shown in Additional file 2.

No significant changes in the mRNA levels were detected in the disruptants of L-PSP (TTHA0137), PIN-domain family protein (TTHA0540) or RNase II (Table 2). TTHA0137 and TTHA0540 are postulated to be RNases based on their sequence motifs. Thus, we may anticipate that the disruption of either of these two genes would have little effect on global mRNA abundance. However, given that *E. coli* RNase II is known to actively degrade transcripts [14], it was surprising that disruption of the RNase II gene had no significant effect on global mRNA abundance in *T. thermophilus* HB8. It should be noted that we did not detect small changes below the significant threshold. In addition, we have conducted a proteome analysis and have detected the presence of RNase II and TTHA0137 in *T. thermophilus* HB8 cells grown in the same growth conditions as used here (K. Kim *et al*., unpublished). These findings also indicate that the RNase R gene was expressed as a protein in wild-type cells under the test conditions used here.

**Table 2:** mRNA abundance changes in the disruptants of each RNase

		**Log phase**		**Stationary phase**	
**Protein name**	**ORF number**	**up**	**down**	**up**	**down**
RNase Y	TTHA1817	37	35	0	0
RNase II	TTHA1534	0	0	0	0
RNase R	TTHA0910	3	11	76	115
PNPase	TTHA1139	19	26	66	88
RNase HI	TTHA1556	116	200	97	137
RNase HII	TTHA0198	74	199	0	0
Argonaute	TTHB068	31	121	110	194
β-CASP family protein	TTHA0252	108	99	38	39
YbeY	TTHA1045	76	69	85	103
PhoH-like protein	TTHA1046	0	0	88	125
L-PSP	TTHA0137	0	0	0	0
PIN-domain protein	TTHA0540	0	0	0	0

The numbers of ORFs with a *q* value <0.05 and a fold change of >2-fold relative to the wild type are indicated in the Table.

Significant changes in mRNA abundance were detected in 7/12 RNase disruptants (in either the log or stationary phases). This analysis showed that the abundance of 905 mRNAs were affected under all the examined conditions for each strain based on our experimental criterion. Of these, 347 were significantly increased in abundance, whereas 503 were significantly reduced in abundance. The increase or decreases in the levels of the remaining 55 mRNAs depended on the growth phase and the specific disruptants. These results suggest that the disruption of RNases affected global mRNA abundance directly by degrading transcripts and via indirect processes. In addition, these data suggest that different RNases act on different substrates and during different growth phases.

### Roles of RNase Y, RNase R, and PNPase in regulating global mRNA abundance

The gene disruptions of RNase Y, RNase R and PNPase, which are known to be involved in mRNA decay [14], resulted in increases and decreases in the abundance of annotated transcripts, but the profiles varied depending on the growth phase. Disruption of the RNase Y gene affected mRNA levels only in the log phase, whereas 37 and 35 genes were up- and down-regulated, respectively (Table 2). These numbers contrasted dramatically with those obtained in the *B. subtilis* RNase Y-depleted strain, where approximately 1,100 transcripts were affected [2]. The most highly up-regulated gene was uridylate kinase (9.1-fold), whereas the most highly down-regulated was TTHB221, a hypothetical protein (0.2-fold). Among the genes that exhibited altered mRNA levels, 17 encoded a transporter or a permease. In addition, 11 genes were related to amino acid transport and metabolism. These results suggest that RNase Y is not the major endo-RNase in *T. thermophilus*, unlike RNase Y in *B. subtilis*.

The effects of RNase R and PNPase gene disruption were more evident during the stationary phase than the log phase. In particular, three and 11 genes were up- and down-regulated during the log phase, respectively (Table 2). This result appeared to be consistent with a report that *E. coli* RNase R affects gene expression during the stationary phase [38]. The genes of the RNase P protein component (TTHA0445) and tRNA methylthiolating enzyme (TTHA1618) were up-regulated in the RNase R gene disruptant. These two proteins are involved in the tRNA maturation process [19,39]. The TTHA1618 gene was also up-regulated during the stationary phase, whereas the SmpB transcript was down-regulated. SmpB promotes RNase R proteolysis by stimulating the binding of Lon and HslUV proteases [40]. The loss of RNase R may result in reduced *smpB* expression. During the stationary phase, 76 and 115 genes were up- and down-regulated, respectively (Table 2). Among the affected genes, 26, 19, 17 and 16 genes were categorized into the COGs codes E, G, I and J, respectively. In the translation category (COGs code J), the genes of five ribosomal proteins, two aminoacyl-tRNA synthetases, and an initiation factor IF-1 were up-regulated. In the lipid metabolism category (COGs code I), the transcripts of two acetyl-CoA acetyltransferases and two acyl-CoA dehydrogenases were down-regulated. Some of these genes are known to be regulated by FadR, which is a TetR-family transcriptional repressor [9]. Another TetR-family repressor, PaaR, was up-regulated, although FadR was not, but these repressors have different target specificities [41]. It is noteworthy that many of the sulfur oxidation (Sox) genes, i.e., TTHA1410 (SoxD) to TTHA1417 (SoxB), exhibited a drastic reduction in their mRNA levels (between 5- and 153-fold). These genes are believed to be involved in oxidative sulfur metabolism [42]. TTHA1414, a putative sulfurtransferase, had the most reduced mRNA level (153-fold) among the RNase R gene disruptant. This effect may be related to the involvement of RNase R in protecting *T. thermophilus* HB8 cells against oxidative stress. Three genes (TTHA0470, TTHA0682 and TTHA1618) were common to the gene lists of the log and stationary phase cells.

The disruption of the PNPase gene (TTHA1139) resulted in 19 and 26 genes with up- and down-regulated mRNA levels during the log phase, respectively (Table 2). During the stationary phase, 66 and 88 genes were up- and down-regulated, respectively (Table 2). The mRNA levels of 10 genes, most of which encode a hypothetical protein, were affected during the log and stationary phase. Among the affected genes in the stationary phase, 16, 11, and 13 genes were categorized into COGs codes E, H, and J, respectively. In the coenzyme metabolism category (COGs code H), genes that encode proteins involved in thiamin biosynthesis, i.e., TTHA0674 (ThiS) to TTHA0680 (ThiD), were strongly down-regulated (2.5- to 20-fold). Similarly, six ribosomal proteins and two elongation factors (COGs code J) were down-regulated only during the stationary phase. This contrasted with the results for the RNase R gene disruptant, which led to the up-regulation of translation-related proteins during the stationary phase (as described above). However, it should be noted that the affected genes were different in the two disruptants.

**Figure 1:**
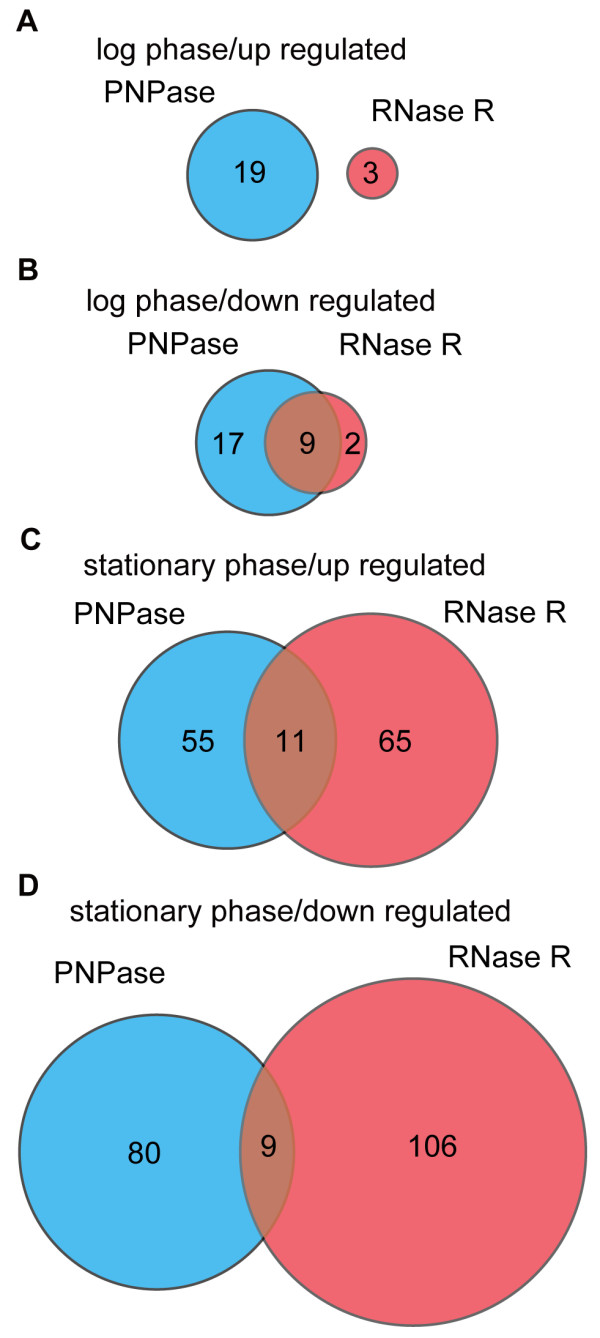
**Venn diagram showing mRNA abundance changes after disruption of the PNPase and RNase R genes.** The cyan and red circles indicate genes affected by PNPase and RNase R disruption, respectively. The numbers in the circles are the numbers of affected genes in the respective areas. **(A)** Genes up-regulated during the log phase. **(B)** Genes down-regulated during the log phase. **(C)** Genes up-regulated during the stationary phase. **(D)** Genes down-regulated during the stationary phase.

Given that RNase R and PNPase are both 3′–5′ exo-RNases, we considered that it would be informative to compare the genes affected by the disruption of these enzymes. Only nine and 20 genes were affected in both strains during the log and stationary phases, respectively (Figure 1). The direction of the change of expression for each specific gene was the same in both strains, where both up- and down-regulation were observed. During the log phase, nine common genes were affected, which corresponded to approximately 20% of the genes affected by PNPase gene disruption. During the stationary phase, 20 common genes were affected, which corresponded to approximately 13% of the genes affected by PNPase gene disruption. The common genes affected in the RNase R and PNPase gene disruptants varied depending on the growth status of the cells. These results suggest that RNase R and PNPase have different substrate selectivity and that endo-RNases act mainly during the log phase, whereas exo-RNase act mainly during the stationary phase.

### Roles of RNase H family proteins in regulating global mRNA abundance

Gene disruption of the RNase H family of proteins, RNase HI, RNase HII and Argonaute, led to changes in the mRNA abundance levels of many genes. This result provided the first experimental evidence that RNase H family proteins are involved in the control of gene transcript levels. The disruption of the RNase HI gene affected global mRNA transcript levels independent of the growth phase, whereas a significant effect of RNase HII gene disruption was observed only during the log phase. In log and stationary phases, RNase HI gene disruption caused the numbers of down-regulated genes (200 and 137,respectively) to be higher than those of up-regulated genes (116 and 97, respectively) (Table 2). In total, the expression levels of 121 common genes (47 up-regulated and 74 down-regulated) were identified as affected during both the log and stationary phases. TTHA0801 and TTHA0802, which encodes a hypothetical protein, were up-regulated dramatically (12- and 35-fold, respectively) during both the growth phases. TTHB012 to TTHB023 were also up-regulated significantly, particularly during the stationary phase (8- and 31-fold, respectively). TTHB023 is a TetR-family transcriptional repressor (PfmR) and is likely to negatively regulate the TTHB018–TTHB023 operon [10]. However, the up-regulation of TTHB023 did not correlate with the down-regulation of this operon. This observation suggests action of another regulatory mechanism. The genes TTHA0976 to TTHA0996 were down-regulated. In particular, TTHA0994 and TTHA0995 were dramatically down-regulated (86- and 1,166-fold, respectively) during both growth phases. TTHA0995 is a novel cyclic di-GMP-degrading protein which comprised a response regulator-domain and a HD-GYP domain [43]. Down-regulation was also observed for TTHA0989 (39- to 75-fold), which encodes a GGDEF-domain protein that is associated with cyclic di-GMP synthetase activity. In addition, most of the genes from TTHA0980 to TTHA0987 were down-regulated by greater than 10-fold after disruption of the RNase HI gene.

Disruption of the RNase HII gene led to the identification of 74 and 199 genes that were up- and down-regulated, respectively, during the log phase (Table 2). The number of down-regulated genes was almost three times the number of up-regulated genes. Among the affected genes, 25, 21, and 21 genes were categorized into COGs codes E, C, and P, respectively. Of these, TTHA1579, a proline dehydrogenase, was the most highly up-regulated gene (6-fold), with concomitant up-regulation of TTHA1577 and TTHA1578, both of which encode enzymes involved in proline and arginine metabolism (COGs code E). The TTHA0995 gene was the most highly down-regulated gene (19-fold) in both the RNase HII and RNase HI gene disruptants. In addition, the 77 genes localized on the megaplasmid pTT27 were affected by the disruption of the RNase HII gene and most (72 genes) were down-regulated. This number corresponds to 31% of the genes encoded on pTT27.

Argonaute gene disruption led to up- and down-regulation of 31 and 121 genes, respectively, during the log phase (Table 2). During the stationary phase, the numbers of up- and down-regulated genes increased to 110 and 193 genes, respectively (Table 2). TTHA0801 and TTHA0802 were up-regulated dramatically (18- and 56-fold, respectively) during both growth phases. During the stationary phase, the genes from TTHB012 to TTHB023 were significantly up-regulated (between 5- and 24-fold). Furthermore, the CRISPR genes (TTHB144, TTHB145, TTHB147–TTHB152, TTHB161–TTHB165, TTHB170, TTHB187–TTHB194, TTHB223–TTHB225, and TTHB230–TTHB231) were down-regulated during both phases. Up-regulation of the genes TTHB012 to TTHB023 was also observed in the RNase HI gene disruptant, as described above. Similarly, TTHA0989 (66-fold), TTHA0994 (2,042-fold), TTHA0995 (177-fold), and most of the genes from TTHA0980 to TTHA0987 (>20-fold) were significantly down-regulated by the disruption of the Argonaute gene, as was the case with the RNase HI gene disruptant. These results suggest some similarity between RNase HI and Argonaute gene disruptants, particularly during the stationary phase. Furthermore, the region from TTHA0976 to TTHA0996 was markedly down-regulated in the Argonaute and RNase HI gene disruptants. It should be noted that the mRNA level of RNase R was increased (2.3-fold) by disruption of the Argonaute gene.

**Figure 2:**
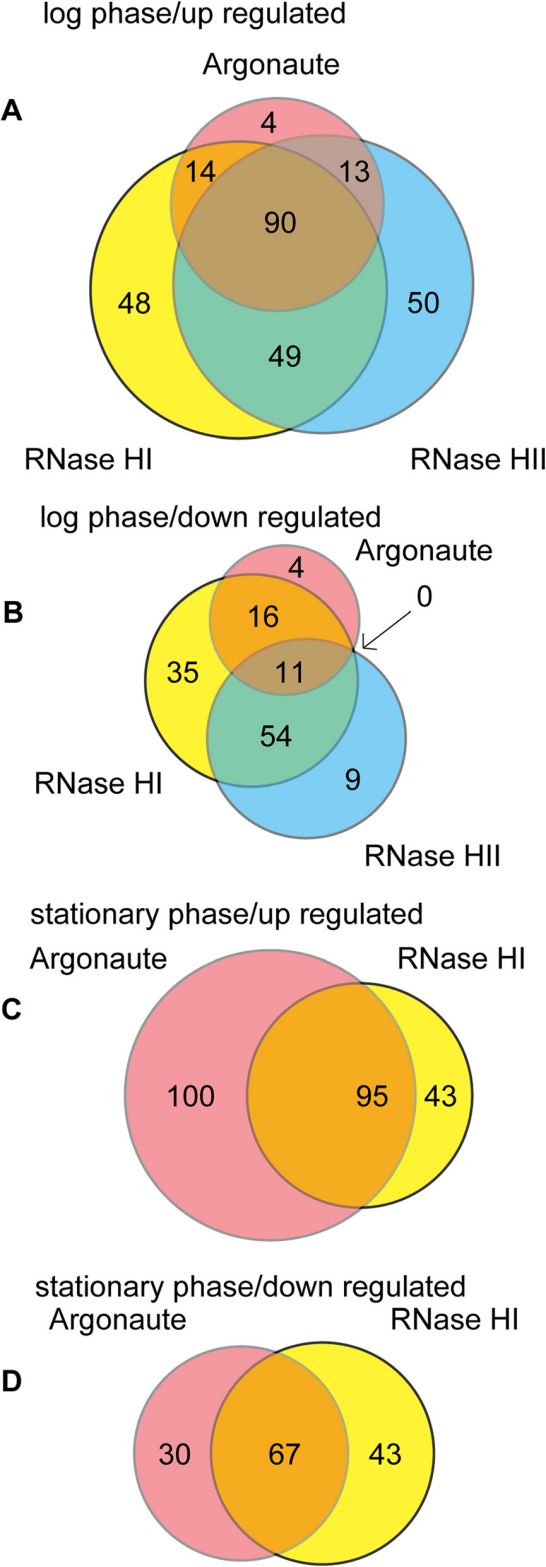
**Venn diagram showing changes in mRNA abundance after the disruption of RNase HI, RNase HII and Argonaute genes*.*** The cyan, magenta and yellow circles indicate genes whose mRNA abundance was affected by disruption of RNase HII, Argonaute, or RNase HI, respectively. Numbers in the circles indicate affected genes in the respective areas. **(A)** Genes up-regulated during the log phase. **(B)** Genes down-regulated during the log phase. **(C)** Genes up-regulated during the stationary phase. **(D)** Genes down-regulated during the stationary phase.

Some genes were commonly affected in the RNase HI, HII and Argonaute gene disruptants. Thus, we compared the changes in mRNA levels among these three RNase H family protein disruptants. As a result, 11 and 90 common genes were up- and down-regulated during the log phase, respectively (Figure 2A). In particular, most of the affected genes in the Argonaute disruptant were also up- or down-regulated in the RNase HI and/or HII gene disruptants. During the stationary phase, 67 and 95 common genes were up- and down-regulated in the RNase HI and Argonaute gene disruptants, respectively (in the RNase HII gene disruptant, no genes exhibited significant change during the stationary phase). Only 23 of the genes affected during the stationary phase exhibited changes in expression in all three disruptants during the log phase, most of which (21 out of 23) were down-regulated. These results suggest that RNase H family proteins, including Argonaute, are involved in regulating the mRNA abundance of specific genes. This gene list included three proteins, i.e., TTHB187 (Cas3), TTHB191 (Cas5e) and TTHB194 (Cas2), and two proteins where the mRNA level increased during phage infection [44]. In the list of genes affected during the log and stationary phases, 24 and 13 genes, respectively, were associated with the CRISPR loci of the megaplasmid and were down-regulated. These results suggest that the RNase H family of proteins is involved in the expression of the CRISPR defense system.

### Roles of putative RNases in the regulation of global mRNA levels

The disruption of the TTHA0252, YbeY, and PhoH-like genes, which are putative RNases, affected many mRNAs (Table 2). Among these, we have already reported the RNase activity of TTHA0252 [6]. In the TTHA0252 gene disruptant, 108 and 99 genes were up- and down-regulated, respectively. During the stationary phase, fewer genes were affected (Table 2) and 40% of genes were similarly influenced during both phases. The total number of genes affected by the disruption of TTHA0252 was greater than that caused by the disruption of the RNase R or PNPase genes. This suggests that TTHA0252 is involved with the global mRNA decay system. In the TTHA0252 gene disruptant, more genes were affected during the log phase than the stationary phase, which contrasts with results obtained with the other disruptants. Moreover, the affected genes were classified into many categories, including COGs codes E, J, C, and P.

For the YbeY gene disruptant cells during the log and stationary phases, the numbers of up-regulated genes (up to 6-fold) were 76 and 85, respectively, whereas the numbers of down-regulated genes (up to 7-fold) were 69 and 103, respectively (Table 2). The numbers of up- and down-regulated genes during both the growth phases were 38 and 37, respectively, which corresponded to approximately half of the genes affected during the log phase. In total, 260 genes were affected by the disruption of YbeY, which suggests the involvement of this putative RNase in global mRNA abundance.

The disruption of the PhoH-like gene, which encodes a putative RNase, resulted in significant changes in mRNA abundance during the stationary phase where 88 and 125 genes were up- and down-regulated, respectively (Table 2). Among the 16 genes that were up-regulated by more than 3-fold in the PhoH-like gene disruptant, seven were also up-regulated (>3-fold) in the YbeY gene disruptant. Similarly, nine genes with more than 3-fold decreased expression were found in both disruptants. The mRNA level of the YbeY gene was down-regulated in the PhoH-like gene disruptant and vice versa. The PhoH-like gene (TTHA1046) is located immediately upstream of the YbeY gene (TTHA1045) in the *T. thermophilus* genome. The disruption of the YbeY gene had similar effects to that of the PhoH-like gene. During the log phase, no significant changes were detected in the PhoH-like gene disruptant.

**Figure 3:**
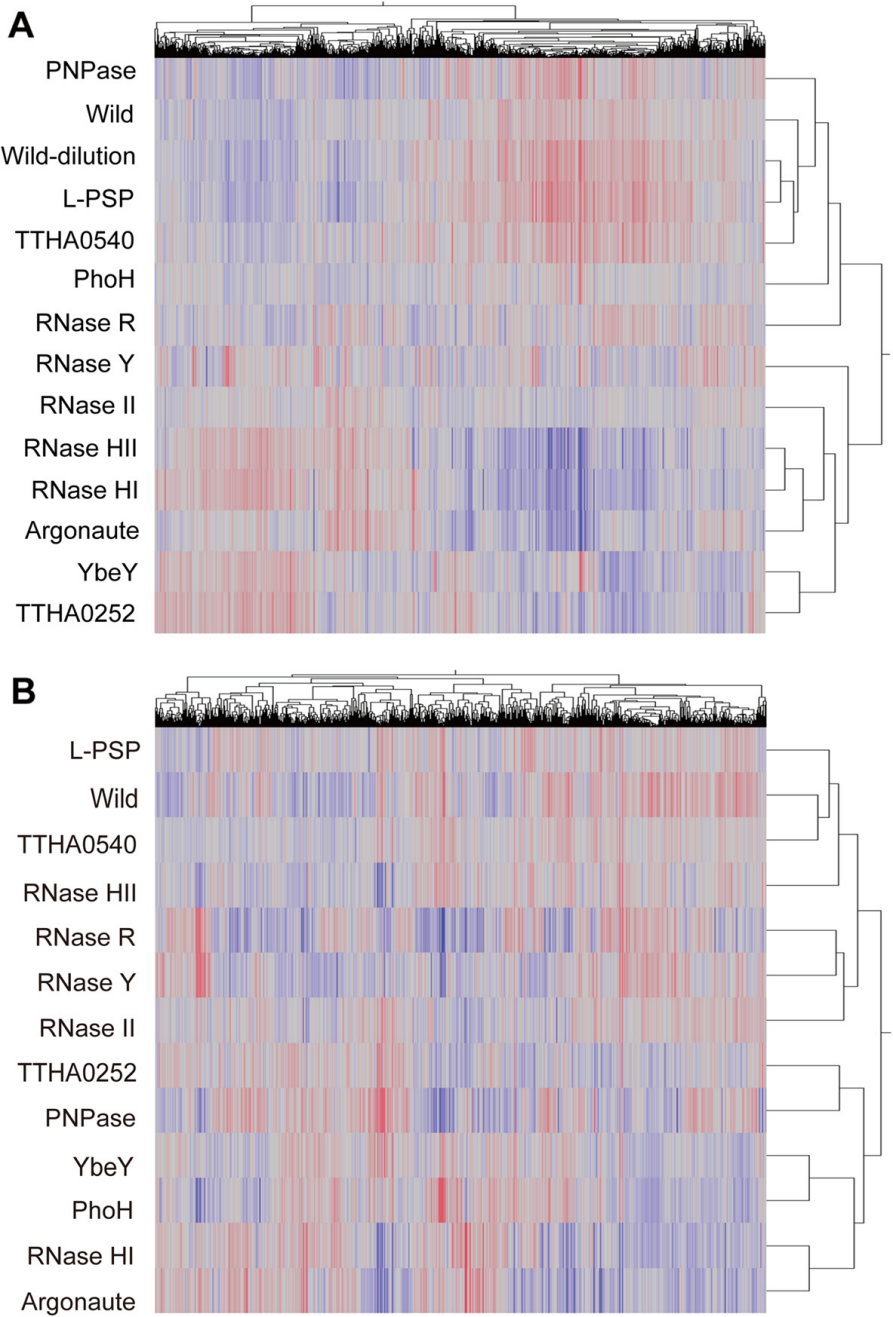
**Tree clustering of the wild-type and disruptants based on the Spearman’s correlation coefficient.** In this two-dimensional graph, the values are color-coded where the scale moves from blue to gray for values between 0 and 1.0, and from gray to red for values between 1.0 and 5.0. The red and blue lines indicate mRNAs with values more or less than the average of all strains, respectively. The gray lines indicate the mRNAs with values close to the average of all strains. **(A)** Log phase. **(B)** Stationary phase. ‘Wild-dilution’ in **(A)** frepresents the sample which was harvested after dilution with hot medium (see Methods).

Comparison of the mRNA abundance changes in the YbeY and PhoH-like gene disruptants showed that 121 common genes were affected, which corresponded to approximately 64% of the genes affected by the disruption of the YbeY gene. During the log phase, the genes affected by disruption of YbeY and TTHA0252 were roughly clustered (Figure 3A). Indeed, 90 of these genes were common and corresponded to approximately 62% of the genes affected by YbeY gene disruption. These results suggest that TTHA0252, YbeY and PhoH-like proteins act as functional RNases in *T. thermophilus* HB8.

### Tree clustering analysis

The changes in the mRNA abundance of some disruptants appeared to be similar, so we performed hierarchical tree clustering analysis using Spearman’s correlation coefficient to group the mRNAs with similar expression profiles (Figure 3). In this analysis, we used the Benjamini and Hochberg false discovery rate (BH-FDR) instead of the *q* value, and we did not use a 2-fold threshold. Therefore, all of the disruptants from both growth phases were included in this analysis. Figure 3 shows that the disruptants of some RNases gene had similar patterns of affected genes, which were clustered together. During the log phase, the patterns of affected genes in the Argonaute, RNase HI, and HII gene disruptants were similar and clustered (Figure 3A), whereas during the stationary phase that of the RNase HII disruptant was not clustered with those of the Argonaute and RNase HI disruptants (Figure 3B). These similarities are also indicated in the results shown in Figure 2. Similarly, the genes with changed mRNA levels during the stationary phase after the disruption of YbeY and PhoH-like genes were roughly clustered (as mentioned above). The RNase R gene transcript level was increased in the Argonaute disruptant, but the disruption of these two genes produced different mRNA abundance patterns according to the tree clustering analysis.

## Discussion

Despite repeated attempts, we were unable to isolate disruptants for the genes that encode RNase J, RNase P, and the nanoRNase, TTTHA0118. *B. subtilis* has two β-CASP family proteins, RNase J1 and J2; RNase J1 is known to be essential [45] but RNase J2 is not [34]. RNase J1 is involved in rRNA maturation [46] and its disruption may affect ribosome assembly. *T. thermophilus* HB8 also has two β-CASP family proteins, RNase J and TTHA0252. RNase J in *T. thermophilus* HB8 may also be related to rRNA maturation, which would make it an essential gene; however, TTHA0252 is not essential and is not an ortholog of RNase J2, but belongs to a different subfamily from RNase J1 and J2. In the present study, disruption of the TTHA0252 gene affected 207 genes during the log phase and 77 genes during the stationary phase. This suggests that TTHA0252 plays an important role in regulating global mRNA levels. TTHA0252 has a 5′–3′ exo-RNase activity and probably has an endo-RNase activity [6]. However, disruption of the RNase J2 gene in *B. subtilis* affected the expression of only 44 genes [47].

RNase P is known to be involved in tRNA maturation. In the absence of the protein component of RNase P, the turnover of RNase P is low because of the slow release of degradation products in low salt conditions [48]. Thus, knockout of the RNase P protein component (TTHA0445) may disrupt tRNA maturation by affecting rapid product release, thereby generating a lethal phenotype. The gene that encodes the protein component of RNase P is also essential in *B. subtilis* and *E. coli*[34].

The RecJ-like protein TTHA0118 specifically degrades nanoRNAs with 5’–3’polarity [21]. Thus, TTHA0118 is a nanoRNase and is believed to be a functional homolog of *E. coli* oligoribonuclease (Orn). In *E. coli*, Orn is an essential protein for cell viability, although the reason for this is unclear. Goldman *et al.* proposed that the toxic effects of nanoRNAs are caused by altered gene expression because of nanoRNA-mediated priming [49]. It should be noted that we successfully disrupted TTHA0118 in a previous study [21]. Recently, it was reported that *T. thermophilus* is a polyploid organism, which harbors multiple genome copies per cell [50]. Therefore, we may not have disrupted all copies of TTHA0118 in our previous study.

The disruptants of three putative RNases gene exhibited no significant changes in mRNA abundance in this study. Among these, RNase II is known to be the main mRNA degradation exo-RNase in *E. coli*[17]. This enzyme degrades RNA fragments with 3′–5′ polarity generated by endo-RNases, such as RNase E and RNase G. However, disruption of the RNase II gene in *T. thermophilus* did not affect mRNA abundance. In contrast, disruption of the PNPase and RNase R genes, other 3′–5′ exo-RNases, led to changes in the mRNA levels of many genes in *T. thermophilus*. These results suggest that RNase II is not a major exo-RNase in *T. thermophilus* HB8. It is possible that the same RNases have different roles in different species.

The disruptants of two putative RNase genes, the L-PSP and PIN-domain protein genes, had no significant effect on mRNA abundance. The rat ortholog L-PSP is known to be an inhibitor of *in vitro* transformation [51] and it also has an RNase activity [23]. Another recent study reported that a L-PSP homolog bound glucose [52]. Thus, we cannot exclude the possibility that TTHA0137 (L-PSP) is not an RNase.

TTHA0540 has a PIN-domain, which is known to have RNase activity, and a TRAM domain, which is predicted to bind RNA. In this study, disruption of the TTHA0540 gene had no significant effects on global mRNA levels. Thus, it is possible that TTHA0540 is not an RNase that degrades endogenous mRNA.

Disruption of the putative RNases genes, YbeY, PhoH-like, and TTHA0252, caused significant changes in mRNA abundance. To the best of our knowledge, this is the first transcriptome analysis of PhoH-like and TTHA0252 orthologs. As discussed earlier, disruption of TTHA0252 caused significant changes in mRNA levels, which suggests that this protein functions as an RNase *in vivo*. In the YbeY gene disruptant, the expression levels of 146 and 190 genes were affected during the log phase and stationary phase, respectively. The structure of YbeY is similar to that of the MID domain of Argonaute, which is a 5′-phosphate-binding domain [29]. Moreover, YbeY is related to rRNA maturation [16]. Therefore, the effect of YbeY gene disruption on global mRNA abundance may be attributable to the loss of its RNase activity. Alternatively, YbeY may affect mRNA levels by interaction with other protein(s) that have RNase activity. The MID domain is also believed to be involved in protein-protein interactions [53]. The YbeY-PhoH-like gene cluster is conserved in most bacteria, including *T. thermophilus*, and an interaction between these two proteins was suggested by a previous large-scale analysis [54]. In addition, YbeY has strong genetic interactions with RNase R and PNPase [28]. Therefore, the effects of YbeY gene disruption on mRNA abundance may be caused by interactions with these RNases. It should be noted that the PhoH-like gene disruptant exhibited no significant changes in the mRNA profile during the log phase, unlike the YbeY gene disruptant. This may indicate that the assumed interaction depends on the growth phase.

In the PhoH-like gene disruptant, the mRNA levels of 213 genes was affected during the stationary phase. Originally, PhoH-like protein was identified as a phosphate starvation-inducible protein, which was subsequently reported to bind ATP [55,56]. A recent sequence analysis suggested that PhoH-like protein resembles the NYN domain, which shares a common protein fold with the nuclease superfamily of proteins [22]. Thus, it is possible that PhoH-like protein binds RNA.

A significant change in mRNA abundance was detected after disruption of the RNase HI and HII genes. This was a slightly unexpected finding because these RNases are believed to function during DNA replication and repair. The RNase H family proteins are DNA/RNA hybrid-specific RNases, which degrade the RNA primers of Okazaki fragments during the DNA replication process. We were able to isolate disruptants of RNase H family genes, indicating that they are not essential genes for cell viability. Thus, the lack of one RNase H family protein may be compensated for by another. Recently, it was reported that RNase HII is involved with the repair of ribonucleotide misincorporation in DNA [57].

*T. thermophilus* RNase HII is a RNA–DNA junction-specific RNase [26]. In particular, this enzyme can only degrade DNA–RNA–DNA/DNA hybrids, and it is believed to generate a nick to eliminate misincorporated nucleoside monophosphate (NMP) from DNA [26]. It is possible that disruption of the RNase HII gene induced DNA instability by the misincorporation of ribonucleotides into DNA.

A significant change in mRNA abundance was detected after disruption of the Argonaute gene, which was similar to that seen after disruption of the RNase HI and RNase HII genes (Figure 3). The structure of the Piwi domain of Argonaute is similar to that of the RNase H domain [14]. This observation suggests that Argonaute may have a similar activity to RNase H family proteins. Moreover, it is known that Argonaute can degrade DNA/RNA hybrid substrates [20]. Thus, Argonaute may be a functional homolog of RNase H. Furthermore, the region from TTHA0976 to TTHA0996 was down-regulated, which suggests that Argonaute and RNase HI are related to the control of this region. Genes of the CRISPR system, a bacterial immunosystem, were down-regulated in the Argonaute gene disruptant. Argonaute may be involved with the immunosystem by regulating the CRISPR system [58].

Finally, the disruption of well-known RNase genes had significant effects on mRNA abundance. RNase Y is believed to be an endo-RNase, which functions during the initial phase of mRNA decay in *B. subtilis*[11]. RNase Y forms the RNA degradosome with RNase J, PNPase and other components [12]. This RNase is essential in *B. subtilis*, but the RNase Y gene could be disrupted in *T. thermophilus*. In addition, although the mRNA levels of 1,104 genes was affected by RNase Y gene knockdown in *B. subtilis*, only 72 genes were affected by RNase Y gene disruption in *T. thermophilus*. This difference suggests that RNase Y is less important in *T. thermophilus* than in *B. subtilis*. In a model of mRNA decay pathways, the endo-RNase activity of RNase Y in *B. subtilis* is believed to take the place of RNase E in *E. coli.* If this endo-RNase activity is essential for mRNA decay, it is possible that there is an alternative to RNase Y in *T. thermophilus*. Intriguingly, no significant changes in mRNA levels were detected during the stationary phase. This may be attributable to the lower expression level of the RNase Y gene during the stationary phase in the wild-type cells (data not shown) or the relatively low contribution of RNase Y to mRNA decay initiation during the stationary phase.

The effects of RNase R and PNPase gene disruption were more evident during the stationary phase than the log phase. RNase R and PNPase are 3′–5′ exo-RNase and cold-inducible RNases in *E. coli*[59]. In particular, RNase R is known to be a stress-inducible RNase [60]. This may explain why the disruption of these RNases gene had a greater effect on mRNA abundance during the stationary phase, compared with the log phase. Furthermore, the level of the RNase R mRNA was increased slightly (1.87- fold) during the stationary phase in *T. thermophilus* and there was an increased level of PNPase mRNA during the stationary phase compared to the log phase.

Another point is that PNPase can polymerize the poly (A) tail of the transcript. In bacteria, mRNA is known to be destabilized by the poly (A) tail [4,61,62]. Thus, polymerization of the poly (A) tail by PNPase could affect the mRNA stability. Thus, the effect of PNPase gene disruption on mRNA levels may have been caused by a deficiency of poly (A) polymerization.

The results of this study suggest that bacterial Argonaute has similar functions to the RNase H family proteins, particularly RNase HI. It is possible that these RNases degrade their substrate RNAs to the same extent. Another possibility is that Argonaute cooperates with RNase H family proteins. Indeed, RNase HI could not fully complement Argonaute gene disruption, and vice versa. Furthermore, Argonaute, the slicer nuclease, is known to be the central component of the eukaryotic RNAi system [63]. *T. thermophilus* Argonaute is known to exhibit a site-specific DNA-guided endoribonuclease activity *in vitro*[20]. It is hypothesized that *T. thermophilus* Argonaute destroys virus or plasmid transcripts directly via its endoribonuclease activity. However, the natural target RNA and the source of the guide DNA molecule(s) for this Argonaute remains to be determined and there is no experimental evidence of its physiological function. Thus, the present study may provide some insights into the function of Argonaute in *T. thermophilus*.

In some disruptants, the number of genes affected depended on the growth phase. It is possible to suppose that the expression levels of RNase genes change according to growth phase; however no significant changes were detected in the RNase gene mRNA levels through growth phase in wild-type strain (Additional file 1). Thus, it is believed that the phase dependence of gene expression was attributable to changes in the abundance of other mRNAs rather than to the functions of RNases. Therefore, the relative activity was changed by increasing or decreasing the levels of mRNAs.

The changes of mRNA abundance observed in this study could be caused by indirect (secondary) effects, for example, disturbed mRNA degradation of transcriptional factors could increase or decrease mRNAs which transcription is regulated by those factors. To investigate this possibility we need to compare the patterns of the transcripts between wild-type and disruptants of transcriptional factors. Furthermore,

## Conclusions

In this study we provided novel experimental evidence for RNase function (Additional file 1). Our study suggests that TTHA0252 and Argonaute are involved in regulating global mRNA levels. This is especially important for bacterial Argonaute, which was previously a protein with unknown function. In addition, our study also suggests that RNase HI and RNase HII have similar function in gene regulation. YbeY and PhoH-like proteins are suggested to act cooperatively as functional RNase. Unexpectedly, RNase Y and RNase II disruption had less impact on mRNA abundance in *T. thermophilus* than in *B. subtilis* and *E. coli*. Moreover, disruption of the RNase Y gene affected mRNA abundance only at the log phase, whereas disruption of RNase R and PNPase genes only had an effect in the stationary phase. These results suggest that the roles of the well-known RNases on global mRNA levels vary in different species and during different growth phases. Finally, this study suggest that *T. thermophilus* HB8 has 13 functional RNases.

## Methods

### Disruption of RNases

The null mutants of *T. thermophilus* HB8 were constructed using a homologous recombination method [64]. The plasmids for gene disruption were derivatives of pGEM-T (Promega, Tokyo, Japan), which were constructed by introducing a thermostable kanamycin nucleotidyltransferase gene (*htk*) [65] flanked by approximately 500 base pairs of DNA up-stream and down-stream of the *target* gene. Disruptants of RNase HI, HII and Argonaute were kind gifts from Dr. N. Ohtani (Keio University).

The wild-type strain of *T. thermophilus* HB8 was cultured in TT medium containing 0.4 mM MgCl_2_ and 0.4 mM CaCl_2_[64]. When the OD_600_ value of the culture reached 0.5, 0.4 ml of the culture was incubated with 1 μg of the plasmid for 4 h to facilitate gene disruption, and the transformants were isolated by positive selection on TT plates (TT medium supplemented with 1.5% Phytagel (Sigma-Aldrich Co., St Louis, MO, USA), 1.5 mM MgCl_2_, and 1.5 mM CaCl_2_) containing 50 μg/ml kanamycin. The deletion of the target gene in the chromosomal DNA was verified using genomic PCR. The sequences of the primers used for genomic PCR are shown in Additional file 3.

### DNA microarray analysis

The *T. thermophilus* HB8 wild-type strain and disruptants were cultured in TT medium at 70°C until OD_600_ reached about 0.8 (the log phase), or about 2.0 (the stationary phase). The log phase-cells were harvested six to eight hours after starting cultivation. The stationary phase-cells were harvested three to six hours after log phase. All mutants showed almost the same final cell density. The RNase R (TTHA0910) gene disruptant had a slower growth rate than wild-type and the other disruptant strains. For TTHA0137, TTHA0540, and TTHA1817 samples (and wild-type) at the log phase, an equal volume of hot medium was added to the culture, and then the mixture was added to an equal volume of 100% methanol and stored at −80°C. For the wild-type and other disruptant strains, the cultures were added to an equal volume of 100% ethanol and stored at −80°C. The total RNA was extracted from these frozen cells according to a previous described procedure [8], except the RNA was resuspended in 20 μl of water after ethanol precipitation. cDNA was synthesized using SuperScript II reverse transcriptase (Invitrogen, Carlsbad, CA, USA) in the presence of the RNase inhibitor SUPERase (Ambion, Austin, TX, USA) and 6-base random primers (Invitrogen). The cDNA was fragmented with 35 units of DNase I (GE Healthcare, Amersham, UK) at 37°C for 10 min. After inactivation at 98°C for 10 min, the cDNA fragments were labeled with GeneChip DNA labeling reagent (Affymetrix, Santa Clara, CA, USA), using terminal transferase, according to the manufacturer's instructions (Affymetrix).

The 3’-terminally labeled cDNA (2 μg) was hybridized to a TTHB8401a520105F GeneChip (Affymetrix) that contained probe sets of 25-mer oligonucleotides representing 2238 ORFs and 1096 intergenic regions. This procedure was basically the same as that previously described, except that 20 μg of herring sperm DNA (Promega) was added to the hybridization mixture [8]. The array was automatically washed and stained with streptavidin-phycoerythrin (Invitrogen) using a GeneChip Fluidics Station 450XP (Affymetrix). The probe array was then scanned with a GeneChip Scanner 3000 (Affymetrix). To determine the mRNA levels in disruptants relative to those in the wild-type, image data of three samples for each disruptant at each growth phase were processed. The DNA microarray assays were performed on biological triplicate samples: RNA was isolated from three independent cultures.

The expression intensity of each of the 2238 ORFs was evaluated using image data and scaled with the one-step Tukey's biweight algorithm using GeneChip Operating Software version 1.0 (Affymetrix). The scaled probe value was calculated as *Sc* = 500 according to the Statistical Algorithms Description Document (Affymetrix). The intensity data sets for each time point were normalized according to the following three steps using the Subio platform basic plug-in program: data transformation (set measurements of less than 1 to 1), per chip normalization (normalization to the 75^th^ percentile), and per gene normalization (using the wild-type log phase or stationary phase data). The false discovery rate (*q* value) [66] of the observed differences in the normalized intensities between the wild type and disruptants was calculated using the BioConductor software package (http://cran.us.r-project.org/).

### Tree clustering

The intensity data sets for each time point were normalized according to the following three steps using the Subio platform basic plug-in program: data transformation (set measurements of less than 1 to 1), per chip normalization (normalization to the 75^th^ percentile), and per gene normalization (normalization to the mean). The false discovery rate (BH-FDR) of the observed differences in the normalized intensities among the wild type and disruptants was calculated using the Subio platform basic plug-in program (one-way ANOVA). Spearman correlation coefficient was used for clustering.

### Availability of supporting data

The DNA microarray data discussed in this study have been deposited in NCBI Gene Expression Omnibus (GEO; http://www.ncbi.nlm.nih.gov/geo/), and are accessible through GEO series accession no. GSE52792.

## Abbreviations

RNase: Ribonuclease; Endo-RNase: Endoribonuclease; Exo-RNase: Exoribonuclease; β-CASP: Metallo-β-lactamase-associated CPSF Artemis SNM1/PSO2; PNPase: Polynucleotide phosphorylase; NYN: Ribonuclease domain; Nedd4-BP1: YacP-like Nuclease ribonuclease domain; KH motif: K Homology motif; L-PSP: liver perchloric acid-soluble protein; CRISPR: Clustered Regularly Interspaced Short Palindromic Repeats; BH-FDR: Benjamini and Hochberg false discovery rate; TRAM: Domain after TRM2 and MiaB domain; PIN domain: PilT-N terminus domain.

## Competing interests

The authors declare that they have no competing interests.

## Authors’ contributions

HO carried out the transcriptome analysis and drafted the manuscript. TS participated in discussion about RNase structure and helped to draft the manuscript. YA participated in the data analysis of transcriptome analysis. KF participated in the transcriptome analysis. NN, AS, RM and SK conceived of the study, and participated in its design and coordination and helped to draft the manuscript. All authors read and approved the final manuscript.

## Supplementary Material

Additional file 1: Figure S1 Putative RNA metabolic pathways in *Thermus thermophilus* HB8. The arrows indicate the flow of the pathways. **Figure S2.** Expression profile of RNase gene in wild-type strain. The profile for respective (putative) RNase gene is indicated as black lines. Red and blue lines represent increased expression and decreased expression during culture, respectively. **Figure S3.** Model of mRNA degradation in *T. thermophilus* HB8 during the log phase. Endo and Exo indicate cleavage via endo-RNase and exo-RNase activity, respectively. **Figure S4.** Model of mRNA degradation in *T. thermophilus* HB8 during the stationary phase. Endo and Exo indicate cleavage via endo-RNase and exo-RNase activity, respectively. Click here for file

Additional file 2: Table S1 Changes of mRNA abundance in each RNase disruptant. This table shows the details of the data presenting in Table 2. Expression indicates the ratio of the mRNA level in the disruptant relative to the wild type. The *q* value indicates the false discovery rate. Annotation for the product indicates the name of the gene product. The COG code indicates annotations of the COG database. Click here for file

Additional file 3: Table S2 Primers used for genomic PCR to assess gene disruption. Click here for file
