# Supplementary material for: The role of ribonucleases in regulating global mRNA levels in the model organism Thermus thermophilus HB8
Source: BMC Genomics. 2014 May 19;15:386. doi: 10.1186/1471-2164-15-386 (PMC4229858; doi:10.1186/1471-2164-15-386)
Supplement: Additional file 1: Figure S1 — Putative RNA metabolic pathways in Thermus thermophilus HB8. The arrows indicate the flow of the pathways. Figure S2. Expression profile of RNase gene in wild-type strain. The profile for respective (putative) RNase gene is indicated as black lines. Red and blue lines represent increased expression and decreased expression during culture, respectively. Figure S3. Model of mRNA degradation in T. thermophilus HB8 during the log phase. Endo and Exo indicate cleavage via endo-RNase and exo-RNase activity, respectively. Figure S4. Model of mRNA degradation in T. thermophilus HB8 during the stationary phase. Endo and Exo indicate cleavage via endo-RNase and exo-RNase activity, respectively. [file 1471-2164-15-386-S1.pptx]

## Slide 1
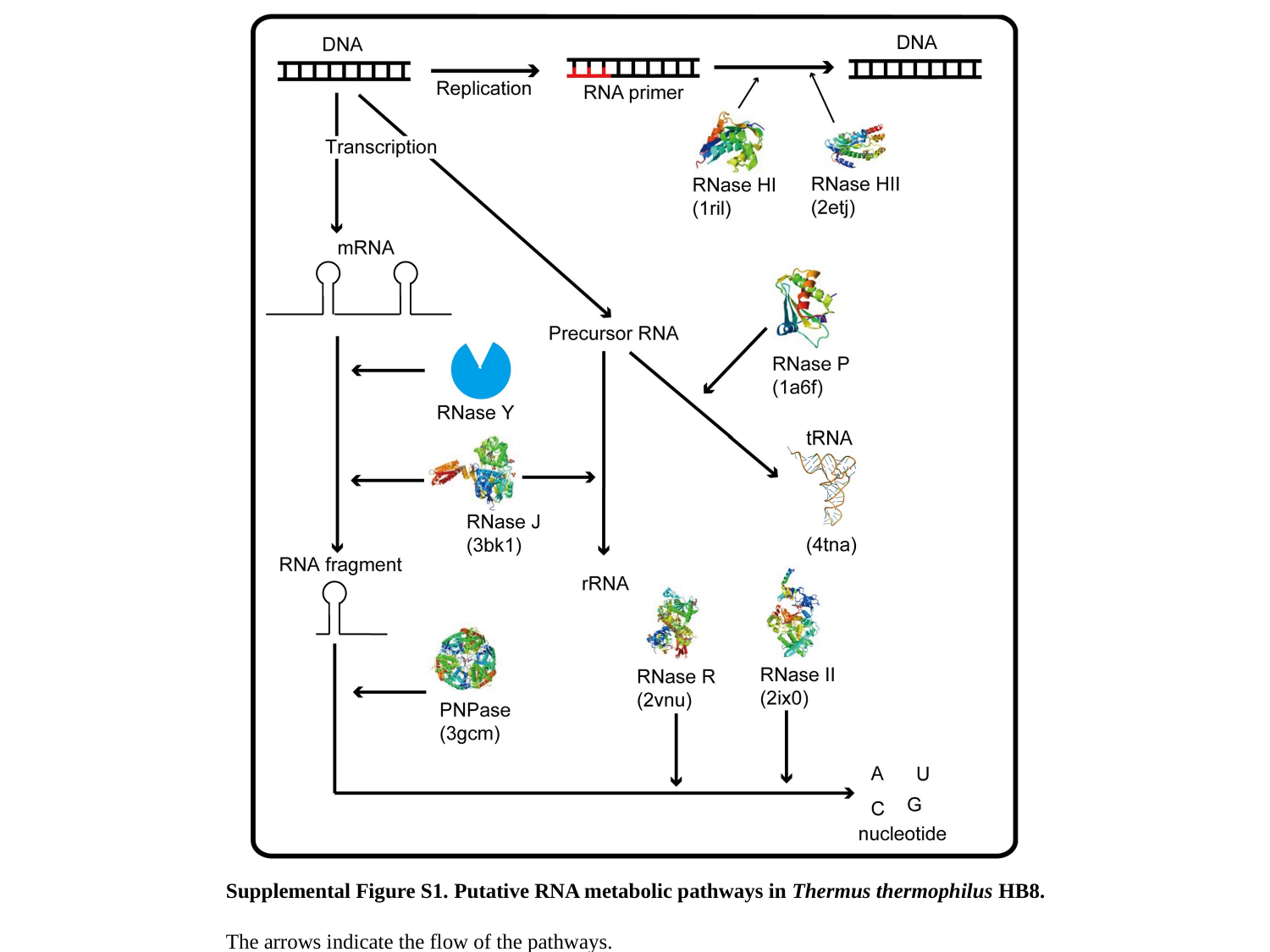

Supplemental Figure S1. Putative RNA metabolic pathways in Thermus thermophilus HB8.
The arrows indicate the flow of the pathways.

## Slide 2
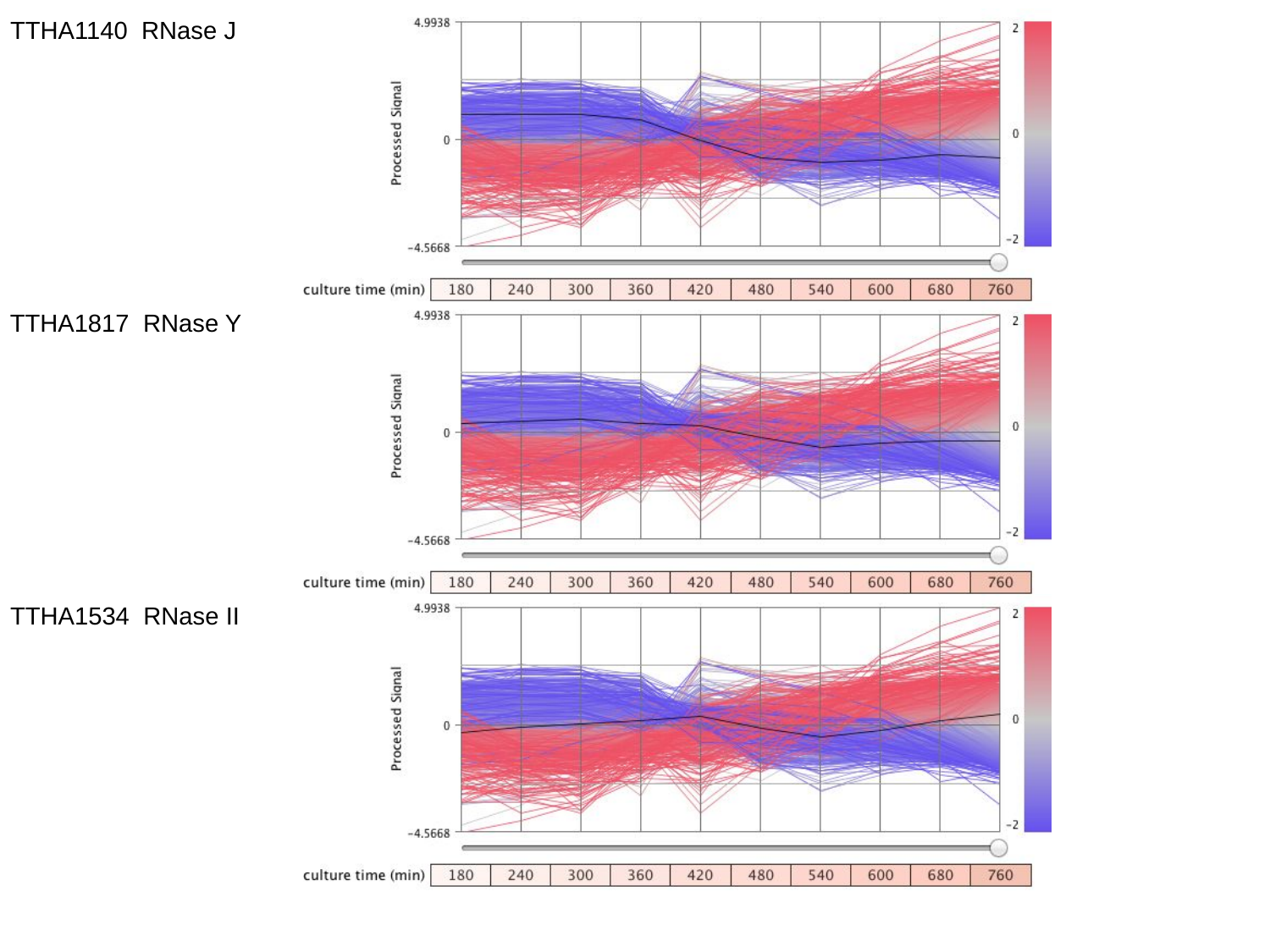

TTHA1140 RNase J
TTHA1817 RNase Y
TTHA1534 RNase II

## Slide 3
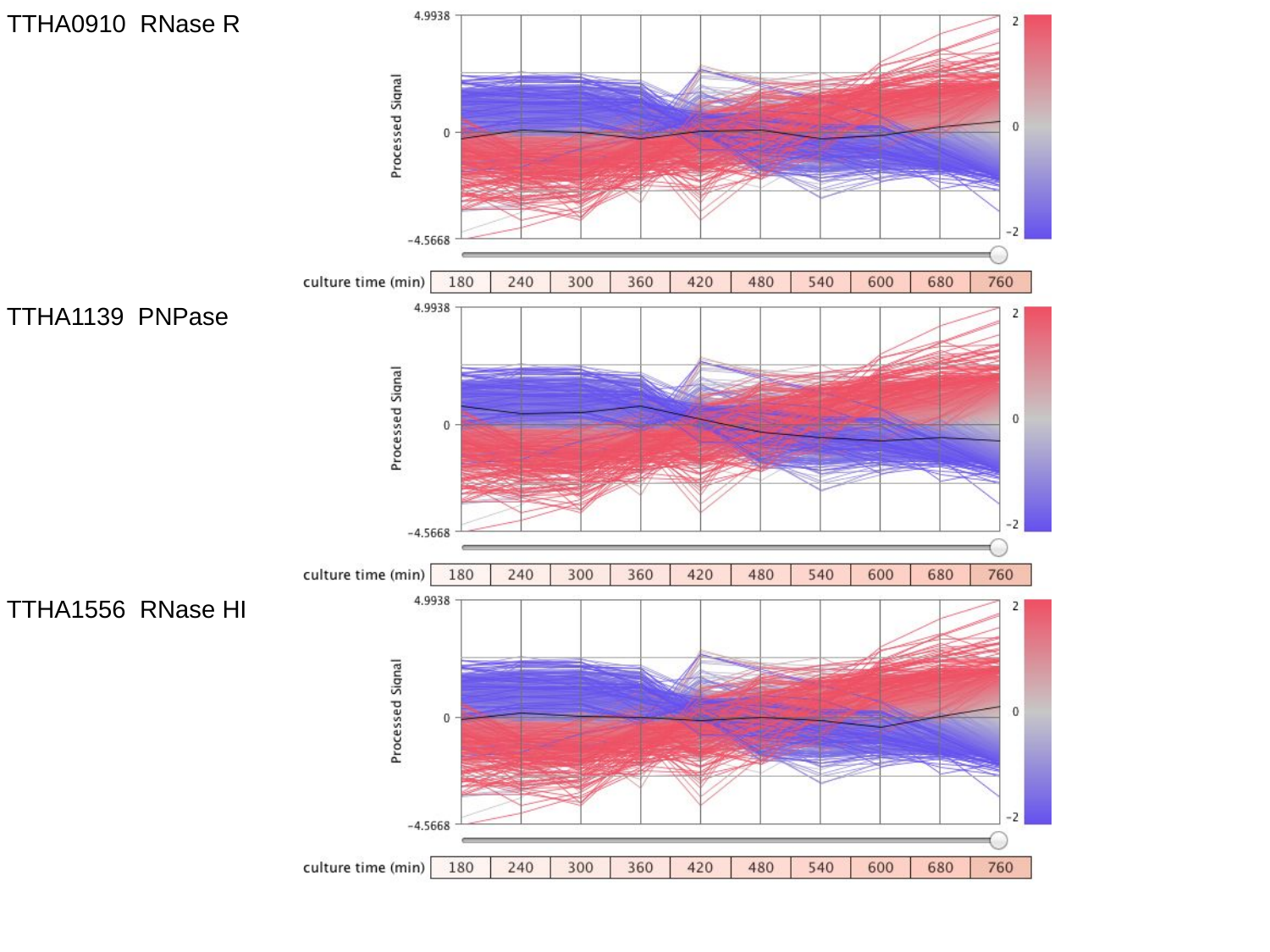

TTHA0910 RNase R
TTHA1139 PNPase
TTHA1556 RNase HI

## Slide 4
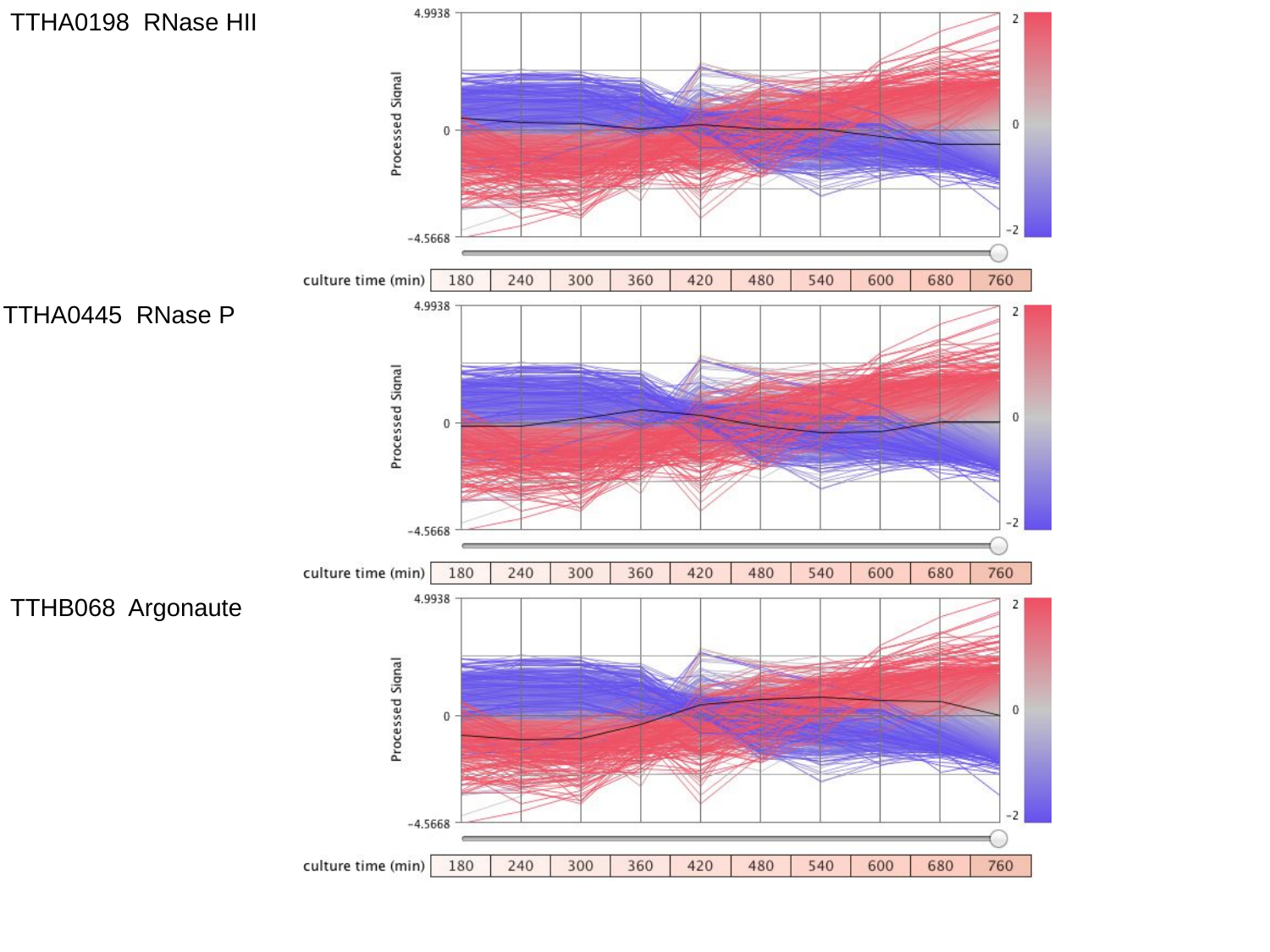

TTHA0198 RNase HII
TTHA0445 RNase P
TTHB068 Argonaute

## Slide 5
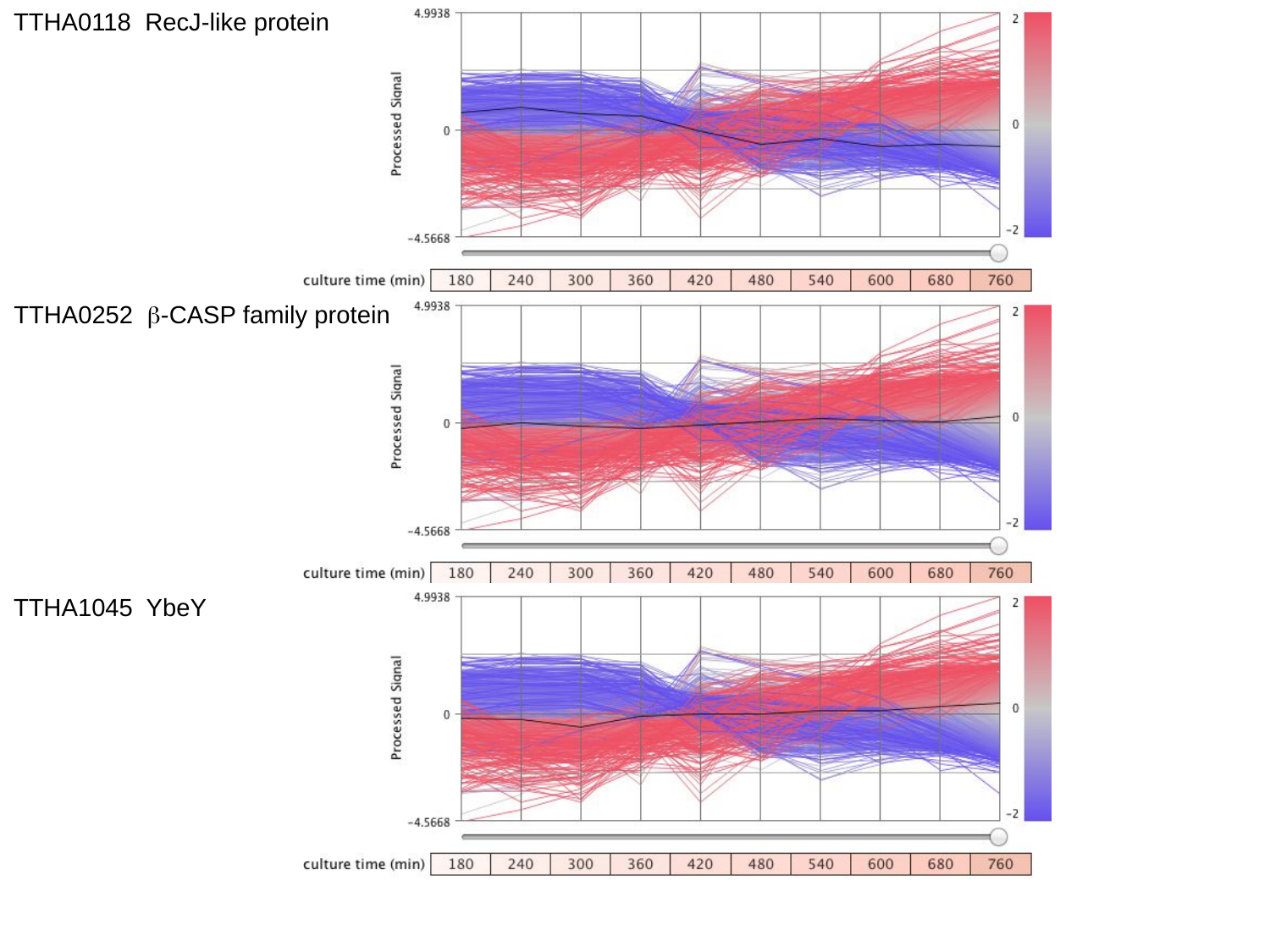

TTHA0118 RecJ-like protein
TTHA0252 b-CASP family protein
TTHA1045 YbeY

## Slide 6
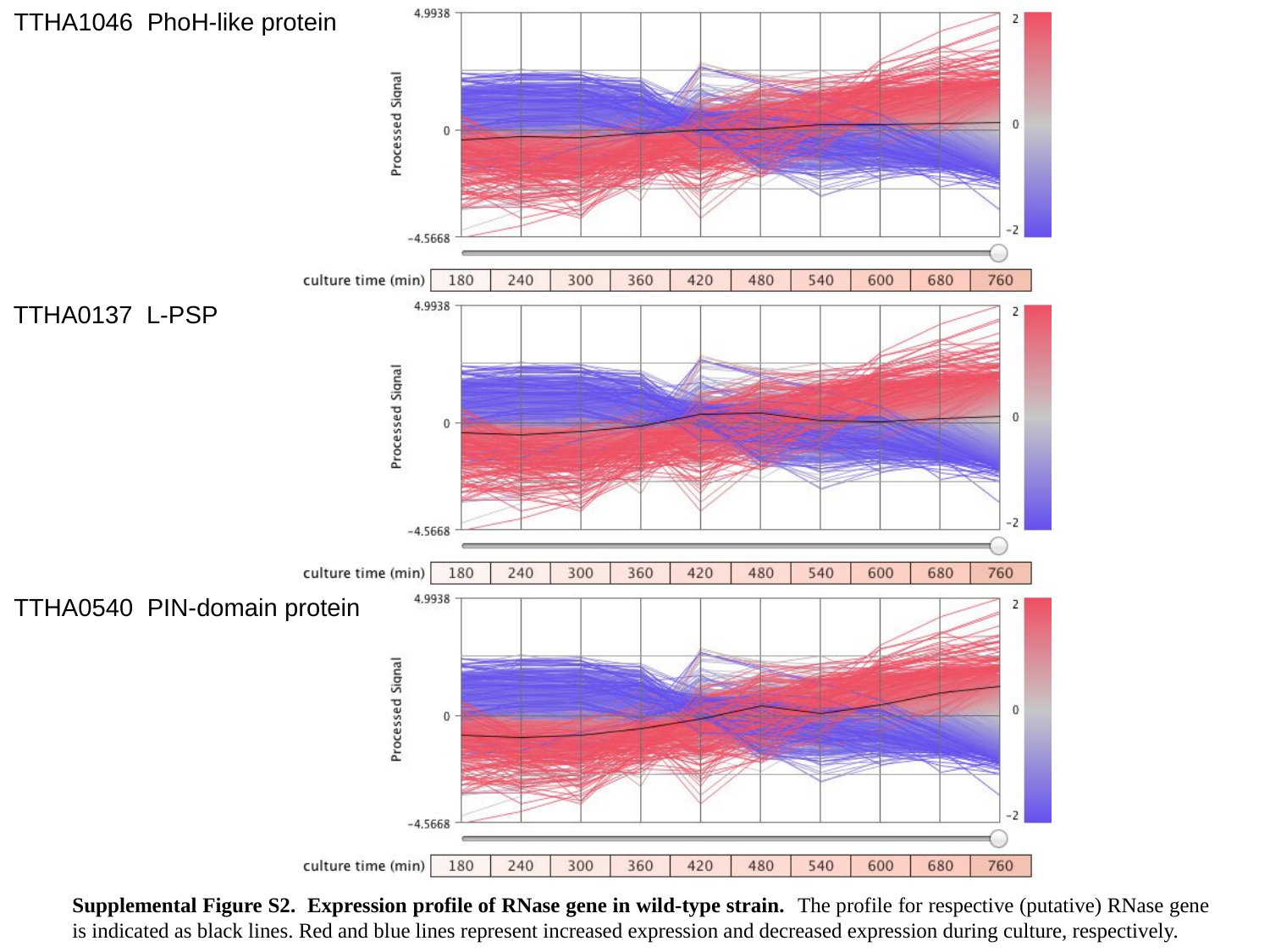

TTHA1046 PhoH-like protein
TTHA0137 L-PSP
TTHA0540 PIN-domain protein
Supplemental Figure S2. Expression profile of RNase gene in wild-type strain. The profile for respective (putative) RNase gene is indicated as black lines. Red and blue lines represent increased expression and decreased expression during culture, respectively.

## Slide 7
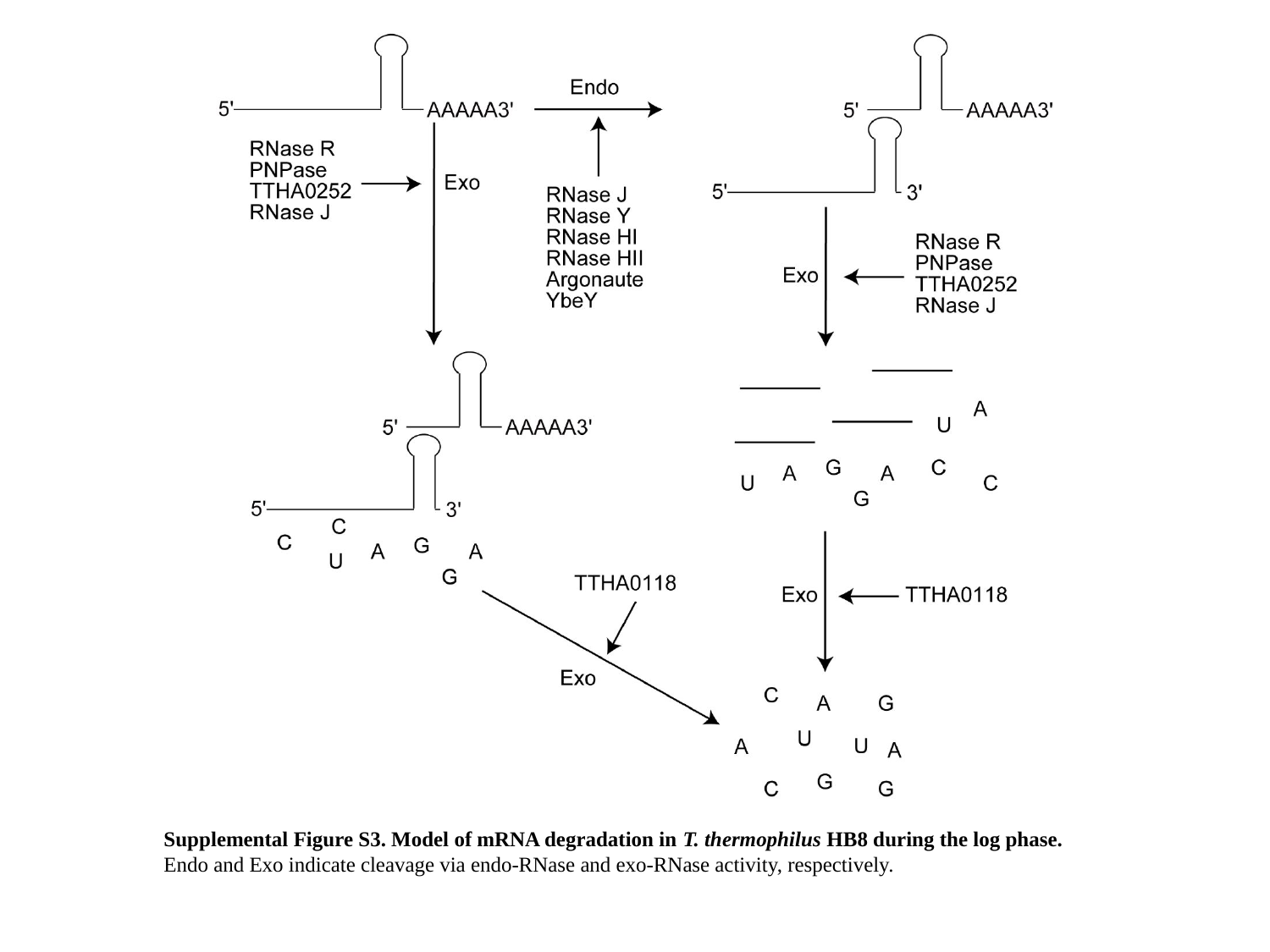

Supplemental Figure S3. Model of mRNA degradation in T. thermophilus HB8 during the log phase. Endo and Exo indicate cleavage via endo-RNase and exo-RNase activity, respectively.

## Slide 8
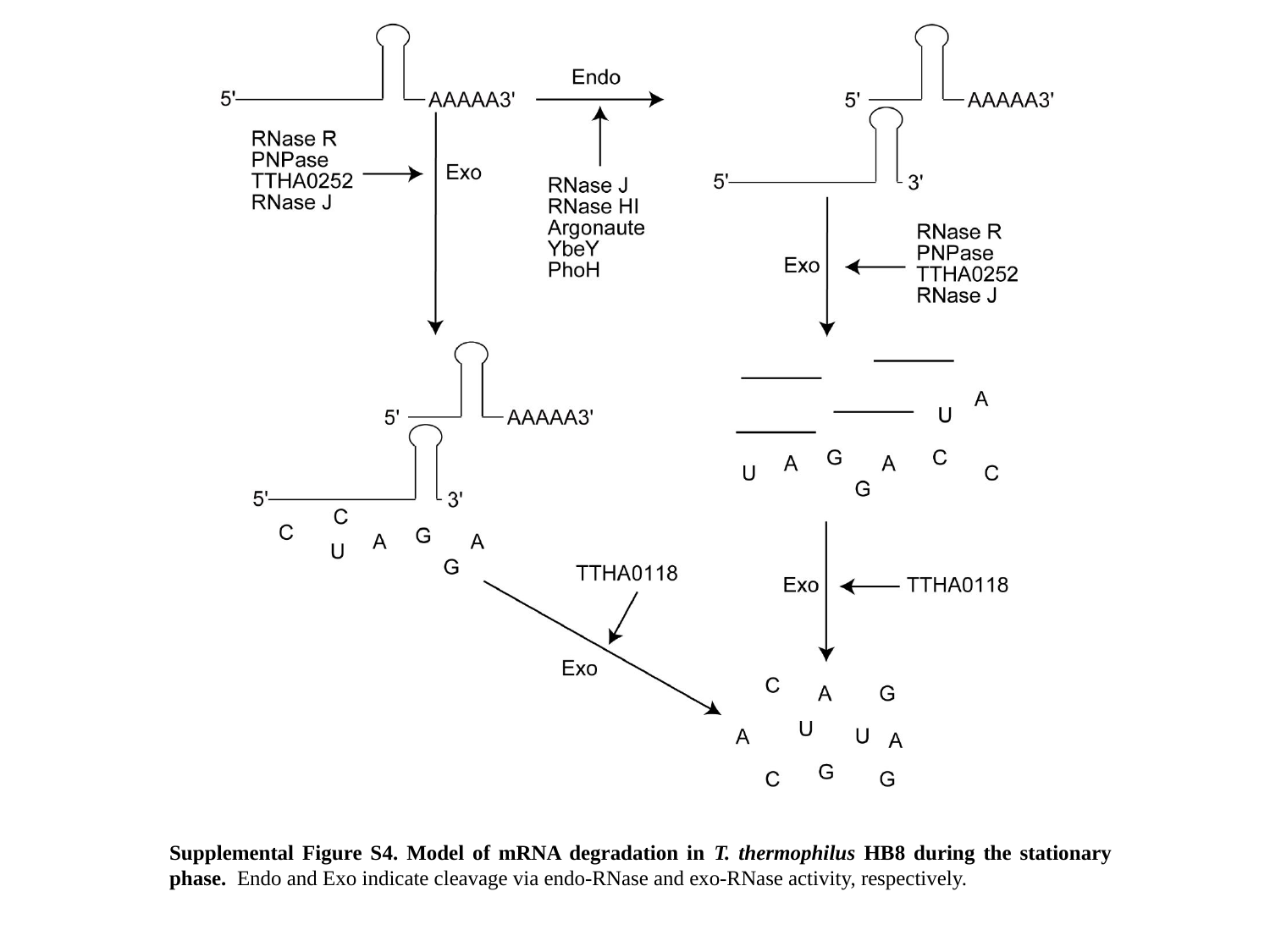

Supplemental Figure S4. Model of mRNA degradation in T. thermophilus HB8 during the stationary phase. Endo and Exo indicate cleavage via endo-RNase and exo-RNase activity, respectively.
